# The IPEDs assemblage: Tracing the entanglements of biomedicine, technology, enhancement and anti-doping policies in sport and society

**DOI:** 10.1177/13634593241306569

**Published:** 2024-12-18

**Authors:** Timothy Piatkowski, Luke Turnock, Nick Gibbs, Cameron Duff

**Affiliations:** Griffith University, Australia; University of Lincoln, England; Northumbria University, England; RMIT University, Australia

**Keywords:** assemblage, drug policy, health, image and performance enhancing drugs, UFC

## Abstract

The US Anti-Doping Agency (USADA) and Ultimate Fighting Championship (UFC) recently ended their anti-doping partnership amidst controversy. We treat this decision, and the motivations underpinning it, as a means of exploring the complexities of anti-doping norms and the blurred lines between image and performance enhancing drug (IPED) use in sport and wider society. Drawing ideas from assemblage thinking, we analyse the evolving power dynamics surrounding IPED use, anti-doping policy, and the role of popular athletes in shaping societal perceptions of the use of, and potential harms associated with IPEDs. The study offers a case analysis of recent controversies in the UFC to investigate the entanglements of biomedicine, technology and celebrity culture in what we call the IPED assemblage. The 2023 termination of the USADA-UFC partnership has sparked debates about shifts in anti-doping standards, raising concerns about weaker testing protocols and perceptions of IPED normalisation. The case of Conor McGregor’s injury recovery and alleged IPED use underscores the blurred lines between therapeutic and enhancement drug use within the IPED assemblage, challenging conventional distinctions between ‘good’ and ‘bad’ drugs in the context of sports management and anti-doping policy making. We highlight the inadequacy of current doping policies in responding to the IPED assemblage and highlight the need to shift public discourse to foster a more critical understanding of therapeutic and enhancement strategies to drive innovation in anti-doping frameworks.

## Background

Scholarly interest in image and performance enhancing drugs (IPEDs) first gained traction in the 1990s (Coomber, 1993, 1999; Kanayama and Pope, 2018), heavily influenced by concerns in competitive sports regarding fairness and the physical risks of these substances. Early research approached the topic from a medical perspective, focusing on the health risks associated with anabolic-androgenic steroids (AAS) and other doping substances (Kanayama and Pope, 2018). Studies highlighted physical harms such as cardiovascular problems, liver damage and hormone disruption (Horwitz et al., 2019), as well as psychological effects like aggression and mood disorders (Pope et al., 2014). In the following decade, research began to diversify as sociologists explored doping beyond elite sports, investigating its prevalence in bodybuilding, fitness, and recreational contexts (Monaghan, 1999, 2002). By the 2000s, scholars began examining the broader social and cultural dimensions of IPED consumption, recognising its role in identity formation and body aesthetics, particularly within subcultures centred on muscularity and fitness (Piatkowski et al., 2020; Underwood, 2017). These developments blurred the boundaries between competitive athletes and those using these drugs for diverse purposes (Hanley Santos and Coomber, 2017; Kimergård and McVeigh, 2014), as more people outside professional sports began experimenting with IPEDs for aesthetic or wellbeing goals. Consequently, contemporary research recognises IPED consumption as a socially mediated and embodied practice that extends far beyond traditional regulatory frameworks, calling for interdisciplinary approaches to understanding its varied use across different social groups (Fomiatti et al., 2019, 2020, 2023; Fraser et al., 2020; Piatkowski et al., 2024b).

Contributing to this call, the current paper draws on Andreasson and Henning’s (2023) definition where IPEDs refer to substances used both inside and outside elite sports to enhance physical appearance, performance, or muscle development. Unlike the strict regulatory framework imposed by the World Anti-Doping Agency (WADA), which targets athletes under the World Anti-Doping Code, the term IPEDs encompasses a wide range of substances and people who use them, including recreational and non-competitive individuals. Reflecting this diversity, we will treat IPED use as an embodied practice influenced by cultural, social, and individual factors, rather than limited to legal definitions or formal anti-doping regulations. While we acknowledge that a strict distinction between competitive and recreational contexts is still common in anti-doping policies globally, like Andreasson and Henning (2023) we are concerned that this separation is more rhetorical than real as IPED use extends into a host of new social contexts (see also Hope et al., 2021). Even so, policies from entities like WADA and United States Anti-Doping Association (USADA), along with sports federations enforcing these regulations through education, testing, and penalties, significantly impact IPED consumption patterns (Boardley et al., 2024). This focus typically aims to maintain competitors’ integrity, health and the fairness of competition, while broader drug policies target societal health and community safety. Despite some overlap, such as recreational drug use in sport, doping remains largely viewed as distinct and morally questionable (Coomber, 1999).

In reality, however, there are significant overlaps between patterns and cultures of drug use in sporting and non-sporting contexts, forcing us to rethink prevailing discourses of health risks, morality, and personal responsibility (Duff, 2004; Piatkowski et al., 2024a; Rhodes, 2009). Though the preference is to treat these contexts separately, discursive framings from the non-sporting world have increasingly bled into sports, shaping doping policies (Andreasson and Henning, 2019; Henning and Andreasson, 2022). Noting this trend, some scholars have observed how drug policies are shaped more by prevailing social attitudes and political contexts – such as the stigmatisation of particular drugs – than by objective decision-making (Gstrein, 2018; Lancaster, 2014). Specific to IPEDs, Beamish (2011) points out how the International Olympic Committee’s stance on these drugs was influenced by entrenched beliefs and social pressures rather than rational argument. Similarly, Coomber (2011) argues that doping policy often reflects broader social fears and myths surrounding drugs in the non-sporting world. He contended that doping policy has evolved by mirroring the discourses used to address illicit drug use, rooted in fear, ‘othering’, and moral panic (Coomber, 2011). This is reflecting in some key doping policy tropes encompassing the dangers of performance enhancement, exaggerated health risks and the notion that doping undermines morality in ways that other forms of cheating do not (Coomber, 2014). Coomber (2014) concludes that this fear-based approach results in policies that fail to adequately protect athletes and often lead to disproportionate and misinformed responses.

The current paper contributes to this discussion through a case analysis of the recent termination of the partnership between the USADA and the Ultimate Fighting Championship (UFC), the leading global brand in mixed martial arts (MMA). The UFC has garnered a substantial international following and significant media attention through its unique blend of sport and entertainment, pioneering the convergence of these industries. Adopting ideas and methods from assemblage thinking, we use this case analysis to trace the interplay of power, policy and public perception in shaping the contemporary landscape of IPED consumption, driven by athletes’ influence and shifting motives for use.

### Approach

The relationship between the UFC and USADA was unique compared to traditional models of cooperation between National Anti-Doping Organizations (NADOs) and sport governing bodies. Unlike typical partnerships where a NADO operates as an independent body enforcing doping controls on behalf of a sport’s governing authority, the UFC and USADA’s agreement was a direct, private partnership (Marrocco, 2015). Established in 2015, this arrangement allowed USADA to oversee the UFC’s anti-doping programme, implementing policies, testing athletes and managing sanctions. The programme was largely funded by the UFC, making it distinct from publicly funded NADO operations like those that govern Olympic and national sports. This financial model inherently introduced a complex power dynamic where USADA’s oversight was financially tied to the UFC, raising questions about autonomy and influence. While this collaboration was praised for bringing rigorous anti-doping measures to UFC athletes, the partnership’s privately funded nature differed from traditional NADO/sport relationships, such as Major League Baseball’s arrangement with the World Anti-Doping Agency (WADA). This underscores the evolving landscape of anti-doping efforts within privately governed sports, where commercial interests and regulatory standards must coexist.

The dissolution of the USADA-UFC partnership presents an opportunity to explore how drug testing and anti-doping policies evolve as sports and entertainment markets intersect. We would argue that these changes further challenge conventional notions of IPED use as morally objectionable or indicative of cheating. With a view to encompassing each of these aspects we will turn to recent accounts of the assemblage and assemblage thinking derived from the work of Gilles Deleuze and Felix Guattari (1987). We propose to treat the ‘IPED assemblage’ to identify, distinguish between, and navigate the diverse forces at work in instances of IPED use in the context of the unfolding USADA-UFC partnership. We contend that the figure of the assemblage provides a basis for mapping relations of power, desire, affect and subjectivity within this evolving partnership, and the ways these relations shape expressions of IPED use both inside and beyond this particular context. Capturing the ways power, desire, force and affect shaped IPEDs across diverse settings and spaces is what we aim to achieve by introducing the idea of the ‘IPED assemblage’ (see also Buchanan, 2020; Duff, 2014). Avoiding starting with the individual, their intentions and proclivities (see Anderson and McFarlane, 2011), assemblage thinking requires us to consider a broader range of forces, structures, material systems, ideas, discourses and relations of power in seeking to make sense of IPED use in particular settings (see also Duff, 2016). Extending the analysis of IPED utilisation to encompass a ‘folding’ (Dewsbury, 2011) of anti-doping policy and sociocultural norms regarding these substances into ‘drug’ assemblages (Duff, 2016), we begin by conceptualising and mapping the ‘IPED assemblage’, drawing in diverse forces and processes in the expression of IPED consumption in a given context, time and setting.

In developing our account of the IPED assemblage, we draw on Duff’s (2023) radical empiricism of the assemblage, which allows us to identify more of the constituent features, component forces, shapes and ‘ends’ of the IPED assemblage through distinctive analytics of power. Duff (2023) contends that assemblages are shaped by unique selection mechanisms (lines of desire, affect and force) which mediate how various elements (bodies, forces, signs, practices) are organised within a given social, political, or organisational structure. By adapting this distinctive analytical approach, we aim to develop new insights into the evolving nature of IPED use and ‘enhancement’ in sports, leisure and recreation. Our approach will indicate how and why new modes of conceptual development and analysis are required to interrogate the ways sports, leisure and recreation are converging, as distinctions between sport and entertainment evolve, along with the changing perceptions of drugs and risk. We seek to challenge conventional views of IPEDs as solely pertaining to performance in competitive contexts like elite sport and underscore the need to conceptualise IPED use within broader societal contexts.

We argue that this kind of analytical and methodological innovation is needed to capture how the UFC is transforming patterns and cultures of IPED use in diverse contexts. The growing popularity of the UFC and the ways this business has addressed IPED use inevitably creates novel power dynamics that shape public perception and policy enforcement. The UFC has exposed tensions between an athlete’s marketability and the applicability of anti-doping regulations, highlighting the ways commercial interests are intersecting with ethical considerations in the enforcement of these regulations. Rather than critiquing the moral force of global anti-doping efforts, our aim is to adopt assemblage thinking to drive fresh insights into the convergence of sports and entertainment markets, with greater sensitivity to the entanglements of power, policy and public perception that frame IPEDs, and the fluidity of regulatory frameworks and the cultural factors influencing their consumption. While we acknowledge that anti-doping policies are shaped by evolving market dynamics, our goal is to highlight how these policies are often misaligned with the realities of their enforcement, rather than challenging their existence or ethical foundation.

## Methodological note

To contextualise the UFC’s evolving relationship with IPEDs, we employ a passive or ‘non-reactive’ digital ethnography (Janetzko, 2008), a modern iteration in digital ethnographic techniques (Pink et al., 2016). This method involves the digital ethnographer collecting and analysing textual and graphical data available online to interpret their meanings (Gibbs and Hall, 2021). Given that much of the UFC’s presence and influence is digitally mediated, this community predominantly communicates and operates online (Pink et al., 2016). Ethnographic immersion took place over the course of October 2023, during which we ‘lurked’ (Gibbs and Piatkowski, 2023), gathering unsolicited textual and graphic content (Enghoff and Aldridge, 2019) from a variety of digital spaces. Social media platforms like YouTube and Instagram were central, as they provided direct insights into the reactions and opinions of fighters, fans and commentators surrounding the UFC’s practices. This included the pages of high-profile MMA fighters themselves, such as Sean O’Malley, Dustin Poirier and others, as well as organisations like *The Mac Life, ESPN MMA* and official *UFC* social media pages, in addition to popular MMA commentary channels like the *Jack Slack* podcast, *Luke Thomas*, the *MMA Guru, MMA on Point* and others. We also monitored news websites such as *The Guardian*, which had covered key developments and specialised martial arts websites such as *Bloody Elbow, MMAFighting, MMA Junkie* and *MMA Mania*, along with forums and blogs where fans and practitioners engage in discussions about UFC policies, IPEDs, and broader industry trends. Special attention was given to discussions surrounding the recent termination of the USADA-UFC partnership, tracing how these developments were discussed across multiple platforms and how these discussions reflected broader concerns regarding performance enhancement, regulatory frameworks and public perception.

Our decision to focus on the UFC, as opposed to other sports organisations, is two-fold. First, with its substantial global following and the high-profile nature of its athletes, the UFC’s case has attracted significant public attention and shaped recent discourse about the role of IPEDs in sports and entertainment. Second, the UFC’s unique position at the intersection of sports and entertainment provides a valuable lens to examine the power dynamics and consequences of IPED use within this industry. Thus, while this analysis focuses on the UFC, it can be read as a broader examination of these themes within the sports and entertainment convergence.

## Findings

### Mapping the IPEDs assemblage

On October 11th, 2023, the US Anti-Doping Agency (USADA) announced that UFC superstar Conor McGregor had re-entered the USADA testing pool after a 2-year absence and that the UFC and USADA had ended their anti-doping programme agreement (USADA, 2023). McGregor’s hiatus followed a severe injury in 2021, leading to speculation about his return and compliance with testing protocols, spurred by his own comments (Baldwin, 2023; Ellis, 2023; Leonard, 2023). Concerns voiced by UFC fighters, along with rhetoric from UFC President Dana White, who in July 2023 dismissed USADA’s 6-month testing pool requirement with ‘Who cares what USADA says?’ were key events that soured the relationship (Tabuenta, 2023; USADA, 2023).

One UFC commentator echoed this, recently declaring that USADA should not oversee the UFC program since we held firm to the six-month rule involving McGregor, and since we do not allow fighters without an approved medical basis to use performance-enhancing drugs like experimental, unapproved peptides or testosterone for healing or injuries simply to get back in the Octagon. (USADA, 2023).

The UFC’s split from USADA, highlighted in Travis Tygart’s statement, offers a unique lens to examine the evolving anti-doping norms amid the merging of global sports and entertainment industries. Tygart mentioned ‘experimental, unapproved peptides’ and testosterone for ‘injury healing’, emphasising medical oversight, which blurs the lines between therapeutic use and performance enhancement (USADA, 2023). UFC Chief Business Officer Hunter Campbell later stressed that discussions about McGregor were purely about medical rehabilitation, not enhancement (The Mac Life, 2023). This raises questions about how the UFC blurs distinctions between sport and entertainment, challenging conventional views on therapeutic versus enhancement substance use. McGregor, one of the UFC’s most popular athletes, generates significant revenue, making his return to the octagon a business decision as much as a competitive one. This suggests the significance of UFC’s commercial interests in this instance, and the perception that the USADA presented barriers to the rapid return of star athletes to competition (or performance). Unlike traditional sport-governing bodies, the UFC operates as an entertainment company, with its anti-doping policies subject to negotiation based on financial and media-driven imperatives. This shift in priorities redefines the purpose of doping regulations from maintaining ‘fair play’ to supporting the UFC’s commercial objectives.

Challenging the longstanding demarcation between ‘therapeutic’ consumption and ‘enhancement’, the UFC’s exit from USADA’s testing protocols underscores the need for new analytical approaches to explore the evolving nature of IPED use in elite sports and its influence on non-elite settings. The UFC’s desire to expedite fighters’ returns to the octagon, underpinned by its entertainment business model, stands in contrast to the principles of long-term athlete health and fairness that traditionally drive anti-doping efforts in other sports. This divergence illustrates shifting power relations, whereby the UFC exerts influence over how anti-doping measures are applied, selectively adhering to or abandoned depending on its business interests. By examining how the UFC may be transforming the IPED assemblage by redefining certain substances as ‘therapeutic’, we can analyse the role of elite athletes’ popularity and status in shaping anti-doping policies and public perspectives on IPED use. Desire shapes the powers of selection that underpin the formation of assemblages (Fox and Alldred, 2022). It establishes connections between elements, granting them new capacities while eliminating others. Within an assemblage, desires can be conscious or unconscious, influencing the selection and arrangement of elements based on individual interests, thus highlighting the complex interplay of productive relations and connections in the ongoing composition of the assemblage (Buchanan, 2020).

We would argue that aspects of this compositional work may be discerned when, at the close of 2023, the UFC flagged an interest in contracting an independent testing agency outside of USADA protocols. What desires does this expression of UFC’s interests, and the broader rejection of USADA policies and testing protocols, reveal or enable within the IPED assemblage? While not an announcement of the removal of drug testing per se, fighter reactions to the termination of the USADA deal treated it as a potential ‘green light’ to use IPEDs, evidenced for example in Demetrius Johnson’s (ONE flyweight champion, former UFC champion) sentiments on social media.

yall bout to start seeing some super athletes lol – Demetrius Johnson (Former UFC champion)

If they tell us we can do it, billy Q abt to get thick afffff. . . but I’ll play by whatever rules we get – Billy Quarantillo (UFC fighter)

The UFC’s shift away from USADA’s testing protocols towards standards perceived as ‘weaker’, such as those of Drug Free Sport, has sparked diverse desires and concerns among fans and fighters. These new standards often lack rigorous out-of-competition testing, leading to perceptions of a more lenient approach to IPED use in the UFC and the broader industry (Lauritzen and Holden, 2023). Raimondi (2023) noted this significant change in a discussion on ESPN.

It is worth noting that Drug Free Sport is a for-profit company, unlike USADA, a non-profit. Drug Free Sport also does not decide who gets tested, how often and when. That will fall on [UFC-appointed “independent administrator” George] Piro, per Campbell and Novitzky.

The termination of the USADA-UFC partnership signals a significant shift in the IPED assemblage, driven by conflicting desires for ‘clean’ competition and rapid returns for popular fighters. This relationship between the UFC and anti-doping bodies shows how commercial and technological imperatives often overshadow traditional regulatory frameworks, resulting in a more flexible and entertainment-driven approach to anti-doping in this context. We now turn our attention to other elements within this IPED assemblage.

### Power and the return to competition: The McGregor controversy

One of the major cited reasons for the dissolution of the USADA-UFC partnership was the situation surrounding Conor McGregor’s return to competition (Martin, 2023). Typically, injured fighters remain in the testing pool even during extended periods of inactivity. McGregor’s three-time opponent Dustin Poirier, commenting on the possibility that McGregor could be granted an ‘exceptional’ return and not be subject to a 6-month testing window before competing, remarked:

if they waive that (six-month testing period) and allow him to compete with no [extended out-of-competition] drug testing, it kind of just makes a joke of the whole thing. Just remove it [testing] completely [at that point]. (cited in Hannoun, 2023)

Moreover, changes in McGregor’s physique during this period of rehabilitation prompted commentary from various sources. UFC bantamweight champion Sean O’Malley, for instance, likened McGregor’s appearance to the ‘Liver King’ (O’Malley, 2022; see Gibbs and Piatkowski, 2023). Similar practices have been observed in the UFC before. For example, Anderson Silva tested positive for AAS following his injury recovery in 2015, and Mirko ‘Cro Cop’ Filipović received a ban for using human growth hormone (hGH) to expedite shoulder recovery (Raimondi, 2015a; Rondina, 2015). The use of IPEDs for injury rehabilitation has thus been established within the UFC well before the McGregor case.

We hasten to add that there is no definitive evidence that McGregor consumed IPEDs to aid his recovery, and we are not making any specific allegations against McGregor in this respect. Rather, we would highlight the speculation surrounding McGregor’s return to the UFC and the spate of cases of UFC athletes commenting on the role of IPEDs in facilitating recovery and thus potentially expediting injured athletes’ return to competition (or their return to work as we have noted already). Indeed, tensions regarding McGregor’s return ought to be considered in relation to these developments, particularly debates about rehabilitation and expedited recovery. The have been numerous suggestions – including from other fighters – that McGregor pulled himself out of the testing pool because of his desire to avoid returning a positive test for banned substances while rehabilitating his injury (Harkness, 2022). Equally, there has been speculation that McGregor held the belief that he deserved an ‘exceptional circumstances’ exemption to normal testing protocols upon his return to the UFC. McGregor’s tweets regarding his withdrawal from the pool and his rehabilitation (cited in Harkness, 2022) were interpreted by many, including Sean O’Malley, as an admission he was ‘*on certain steroids or something to heal the bone*’ (O’Malley, 2022):

‘The [percent] of the bones joining back after a break like this is so low. You think I give a fuck about anything else. . .’ ‘everything was fully disclosed before I began’. – Conor McGregor (cited in MMA Fighting, 2022)

With USADA specifically highlighting their concerns about the use of ‘*performance-enhancing drugs like experimental, unapproved peptides or testosterone for healing or injuries*’ as a major point of contention in their relationship with the UFC, McGregor’s statements on social media appeared to confirm fan speculation that this could be the reason McGregor was withdrawn from the testing pool. The suggestion that McGregor was allowed to evade normal testing procedures when other athletes, such as Mirko Cro Cop, were not given such latitude, has only amplified the perception among some fans that there is a two-tiered system operating with regards to testing in the UFC and how the outcomes of tests are treated, where the ‘superstar money-makers’ are to some extent ‘above the rules’ (e.g. The MMA Guru, 2023).

While such claims of preferential treatment for celebrity athletes are not new (being a common complaint e.g. in professional cycling), what makes the McGregor case distinct within the IPED assemblage is the UFC’s overt shift away from established anti-doping norms. The UFC’s split with USADA signals a structural shift in how the UFC itself engages with IPED regulation, particularly in framing certain IPEDs as ‘therapeutic’ rather than purely performance-enhancing. Unlike past cases, where doping issues were handled discreetly or behind closed doors, the McGregor controversy unfolded at a time when the UFC’s commercial interests and entertainment value dramatically undermined the distinction between competitive sport and commercial spectacle. This makes McGregor’s case part of a broader systemic transformation, where IPED use may be openly justified as part of the ‘entertainer’s’ recovery process, thus challenging the fundamental anti-doping logic in sports.

Conor McGregor’s withdrawal from the USADA testing pool and subsequent speculation about his recovery strategies illustrate the complex interplay of power, desire, money and fame within the IPED assemblage. McGregor’s case highlights the contentious issues surrounding anti-doping policies, especially for high-profile athletes and exemplifies the value of ‘assemblage thinking’ in examining IPED use. This approach considers both formal regulations and dynamic relations of power and popularity that transform the meaning and status of IPEDs. The McGregor case reveals how commercial pressures, the athlete’s celebrity and technological advancements in medicine intersect, revealing tensions in the governance of IPEDs in ways that go beyond the mere evasion of anti-doping rules. McGregor’s situation may reinforce perceptions of preferential treatment for famous athletes or suggest that IPEDs are being redefined as therapeutic tools aiding recovery. The commentary surrounding his case reflects both interpretations, indicating McGregor’s potential preferential treatment due to his celebrity or his use of expensive, experimental treatments for injury recovery. This underscores how the IPED assemblage rapidly evolves as sports and entertainment converge, particularly in industries like MMA and the UFC.

### Blurring boundaries of enhancement and ‘therapy’

As Conrad and Potter (2004) argue, pharmaceuticals often transition from therapeutic treatments to enhancement tools in non-clinical contexts. Their examination of hGH reveals three intertwined facets of biomedical enhancement: normalisation, restoration, and performance enhancement. Likewise, the McGregor case illustrates how substances oscillate between restoration/recovery and enhancement/cheating. As desire, money, celebrity culture, global entertainment brands and social media transform the IPED assemblage, this oscillation intensifies, altering the identities of IPEDs, shifting perceptions, and changing attitudes towards their use.

Of course, we acknowledge that the enhancement/therapy divide is not new. Various high-profile athletes have publicly received stem cell or other advanced treatments (Cash, 2023; Matthews and Cuchiara, 2014) including experimental peptides (Dow, 2014; The Guardian, 2016), highlighting how the boundaries of what constitutes acceptable medical intervention versus performance enhancement are continually evolving. The significance of this UFC case context lies in its potential to shift societal understanding of enhancement in ways that surpass other forms of athletic performance enhancement, as celebrity athletes often set trends that filter down into public consciousness and ‘lay’ behaviours. Indeed, speculation surrounding McGregor’s alleged attempts to evade testing rules and potential facilitation by the UFC has sparked discussions about a perceived ‘two-tier’ system for athletes within the UFC. However, more importantly, McGregor’s case underscores the evolving boundaries between enhancement and therapeutic drug use within the IPED assemblage. While this case brings attention to broader, long-standing critiques about the therapy/enhancement divide in sports, it further complicates this picture by positioning this rhetorical divide within the specific entertainment and commercial structures of global sports. This shift is driven by the extended capacities of IPEDs beyond their immediate consumption events, influencing the changing meanings attributed to their use in various contexts (Piatkowski et al., 2023). Hunter Campbell’s insights into McGregor’s case, as discussed in response to USADA’s statement, illuminate this evolving process.

The conversations I had with Conor and his physician when [the leg break] occurred had nothing to do with fighting. They were legitimately concerned they weren’t going to regain full use of his leg ever again, including the ligaments around it. (The Mac Life, 2023)

Here is an event in which the meaning and status of IPEDs are each in flux, in which distinctions between ‘treatment’ and ‘enhancement’ become uncertain, pointing to wider debates across professional competitions as global sports have evolved into global businesses. Professional athletes, who inherently push the boundaries of human performance, inevitably face injuries (Overbye et al., 2013; Piatkowski et al., 2023) that necessitate medical intervention. Some of these medical treatments have the potential to enhance performance by facilitating growth and repair, in ways that may be perceived as affording an unfair advantage, even as their therapeutic utility is acknowledged. Attempting to accommodate this complexity, WADA has acknowledged the use of drugs for legitimate medical purposes, even if that use could be considered as performance ‘enhancing’ in certain circumstances (WADA, 2023), by establishing ‘therapeutic use exemptions’ (TUEs), which grant athletes permission to use certain substances when a valid medical need exists. Nevertheless, TUEs have proven to be controversial mainly because of the criteria used to distinguish between ‘therapeutic’ and ‘enhancing’ treatments. Athletes, for example, have raised concerns regarding cases where they were denied TUEs for in-competition use of a medicine that they were authorised to take during training (Okamoto, 2015; Rondina, 2015). This issue is mirrored in the discussions surrounding substances like tramadol, where debates about its therapeutic use versus potential for abuse have long been documented (Zandonai et al., 2021).

In the realm of evolving medical technologies, Conor McGregor’s case highlights the increasingly blurred distinction between ‘unacceptable cheating’ and ‘legitimate medicine’. Another UFC instance involves TJ Dillashaw and Cody Garbrandt’s bantamweight title rivalry. Garbrandt sought stem cell treatment in Germany to speed recovery from a back injury under UFC President Dana White’s recommendation (Hiergesell, 2019). Stem cell therapies, despite lacking FDA approval as noted by USADA guidelines (USADA, 2016), were not considered cheating at the time, contrasting with the controversy over McGregor’s alleged use of peptides for injury recovery (Sarmah, 2023). The complexity of this issue is echoed in the context of asthma treatments, where salbutamol usage among athletes has also been scrutinised, raising questions about the balance between therapeutic needs and performance advantages (Allen et al., 2019). This raises further questions about the acceptance and regulation of therapeutic treatments in sports, especially regarding their potential performance-enhancing effects (Turnock, 2022, 2024; Woolf et al., 2021). In the framework of assemblage thinking, the differential treatment of therapies like research peptides and stem cells in sports may stem from broader systems of power and privilege embedded in social structures. Research peptides are widely available at low cost from online suppliers, often marketed as veterinary medicines or research compounds. In contrast, stem cells are accessible only through specialised clinics at considerable expense, limiting access primarily to those who can afford such exorbitant costs. This disparity underscores and exacerbates existing inequalities within sports, mirroring broader societal disparities.

Extending then the logic of the assemblage thinking adopted throughout this article, we would further suggest that the desire for these therapeutic interventions, be it IPEDs such as peptides or stem cells, should be understood not merely as isolated subjective intentions but as affective functions of encounters within an assemblage of ‘forces’ (Duff, 2014: 142–148). One potent example of these ‘extra-subjective’ forces, highlighted in our analysis above, is the labelling and categorisation of which treatments are permissible or impermissible within a particular context like UFC, and the ways these processes inevitably begin to mediate relations of the permissible and the impermissible in other contexts. While actors within the UFC are constrained by particular structural forces, they are also afforded opportunities according to their status and wealth, such as the opportunity to access experimental stem cell treatment in exclusive clinics in Germany. By appearing to tailor the regulation of fighters’ treatment regimes according to relations of accessibility and privilege, rather than uniform and consistent policies like those WADA and the USADA stipulate, the UFC is arguably contributing to the ongoing disruption of the IPED assemblage in ways that appear to be transforming IPEDs into therapeutic tools as opposed to means of unfairly enhancing performance. Our conjecture here, which ought to be considered as a hypothesis to be further tested rather than an established contention, is that approaches to IPEDs across the UFC are influencing attitudes towards these substances in other, non-elite, contexts. Of particular note is the ways IPEDs are being used, talked about and justified in relation to the facilitation of recovery from significant injury rather than performance enhancement per se.

On this point, there is another interesting aspect of Garbrandt’s case worth highlighting, in relation to perceptions of ‘cheating’ and the values that ground or disrupt these perceptions. Despite Garbrandt openly receiving stem cell injections in an attempt to ‘make’ the 2017 title fight against T.J. Dillashaw, when his opponent later tested positive for erythropoietin (EPO) following an unsuccessful bid to become champion a weight class lower, Garbrandt reacted by calling Dillashaw. a ‘cheating bastard’ (Guillen, 2022). In Dillashaw’s justification for using EPO, which he admits was a case of him knowingly using a prohibited substance, it is interesting that he framed this behaviour in terms of his desire to recover from the negative impacts of the drastic weight loss required for him to drop down to 125lbs for his fight with Henry Cejudo:

I pushed my body to the extreme. . . About six weeks out, my body started to crash, it started to get tired. I started feeling like I didn’t want to get up for practice. – T. J. Dillashaw, cited in Raimondi (2019)

I wanted something so bad. . . and the real fucked up part, and again, I’m not creating an excuse – I fucked up, I did it – is the fact that **I never took my body to anything that was un-normal**. I walk around about 45 [hematocrit level]. The highest I ever got my hematocrit back to [on Epo] was 46. It wasn’t like I was above natural levels. The highest a human body can even go naturally is 50. T. J. Dillashaw, cited in Marrocco (2019) (our emphasis)

In a 2019 interview with Dillashaw, former UFC fighter Chael Sonnen reported that he was prescribed EPO (‘Procrit’) by a physician for anaemia during a training camp (Sonnen, 2019). Despite knowing it was a banned substance and that it would have a performance-enhancing effect, this compound was acquired under medical guidance, for a recognised medical condition and was used for this purpose. Yet the use of this was treated starkly differently to a stem cell treatment that undoubtedly allows athletes to recover faster and return to training earlier – one of the main benefits athletes gain from their use of IPEDs (Piatkowski et al., 2023; Turnock, 2022, 2024). We note that some time ago Coomber (1999) described how the categorisation of a drug as beneficial or harmful is often influenced by vague reasoning and moral assessments that depend on shifting definitions of what constitutes ‘medicine’ and the ‘misuse’ of medicine at a given time. Something very similar appears to be happening within the IPED assemblage as the categories of permissible and impermissible substances shift and morph according to the dynamics of power, privilege, celebrity and profit. This analysis calls into question the confidence with which scholars and policy makers have long sought to distinguish between ‘performance enhancement’ and ‘therapeutic interventions’ by highlighting how context-dependent these distinctions so often are. In drawing from recent examples taken from the UFC we have sought to examine how these context dependencies work within the IPED assemblage, as different forces shape the ways certain substances may morph into therapeutic tools for aiding recovery and a return to competition/work in certain circumstances, even as they remain condemned in others.

## Conclusions

The examination of relations of power and desire (Duff, 2023) within the IPED assemblage offers a transformative perspective for exploring the contentious place of drugs in sport. We would argue that the case of the UFC indicates that established distinctions between ‘enhancement’ and ‘therapy’, ‘cheating’ and ‘recovery’ – or even the permissible and the impermissible – are breaking down in ways that have significant implications for anti-doping policies globally. Ongoing debates in the sport and ethics literature (see Coomber, 2014, 2023; Henning et al., 2021; Henning and Dimeo, 2015; van de Ven, 2016; Gibbs et al., 2024) confirm just how contentious these issues are. Some scholars have proposed policy changes that differentiate between substances with minimal enhancement benefits and traditional doping violations, while also allowing provisions for recreational athletes outside competitive contexts.

As sport and entertainment merge in global businesses like the UFC, traditional notions of fair competition are becoming less stable, and the differentiation between ‘clean’ athletes and ‘cheats’ is increasingly blurred. Emerging events like *The Enhanced Games*, which advocate for unrestricted performance enhancement, reflect how these changing views are gaining traction, pushing the boundaries of what is considered fair competition and questioning long-standing anti-doping norms (see Richardson, 2024). Issues of recovery and injury management surely evolve too, as individuals evolve from athletes to entertainers eager to return to work. We are not justifying experimental IPED use or suggesting anti-doping efforts be abandoned. Instead, we argue that these shifting contexts call for new perspectives, like the assemblage thinking presented here, to rethink the role of drugs in sport.

As yesterday’s experimental treatment becomes today’s routine supplement, we are reminded of the innately unstable character of the categories of ‘drugs’, ‘medicines’, ‘treatments’ and ‘poisons’ (Tupper, 2012). The controversies that arise in attempts to codify and stabilise these categories in sporting contexts reflect ongoing debates, particularly the contrasting perceptions of ‘cheating’ and ‘legitimate medicine’. Anti-doping policies are struggling to keep pace with evolving approaches in elite sport, where experimental treatments are becoming mainstream. As yesterday’s controversies turn into accepted practices, perceptions of risk, reward and safety are shifting, influencing attitudes and behaviours beyond elite sport. By acknowledging that doping policy is influenced by broader drug policy discourses, we can appreciate the complexity of these interrelationships and advocate for policies that prioritise athlete well-being over moralistic fears. We cannot offer solutions to these challenges at this point, but we call attention to the value of understanding how IPEDs are evolving in different global contexts to generate a more balanced and informed perspective on the diverse forces at play. Such understandings must consider the complexity of current debates in sport and ethics literature, which should help frame what the next generation of anti-doping policies entails. Ultimately, a meaningful shift in doping discourse and policy could have the potential to influence drug policy more broadly, fostering a healthier relationship between athletes and their choices.
